# Association Between Polypharmacy and Socioeconomic and Demographic Factors in Adults Aged 50 Years and Older by Brazilian Macroregions

**DOI:** 10.1002/pds.70307

**Published:** 2026-01-30

**Authors:** Orlando Luiz Do Amaral Juniot, Thiago André Carniel, Vanessa da Silva Corralo, Fátima Kremer Ferretti, Clodoaldo Antônio De Sá

**Affiliations:** ^1^ Graduate Program in Dental Sciences Universidade Federal de Santa Maria Santa Maria Brazil; ^2^ Graduate Program in Health Sciences Universidade Comunitária da Região de Chapecó Chapecó Brazil

**Keywords:** healthcare disparities, polypharmacy, socioeconomic factors

## Abstract

**Purpose:**

This study aimed to investigate the proportions of polypharmacy in the macroregions of Brazil, considering socioeconomic and demographic factors and their associations.

**Methods:**

A cross‐sectional analysis was conducted using data from the second wave (2019–2021) of ELSI‐Brazil. The outcome was self‐reported polypharmacy. Independent variables included sociodemographic, health, and behavioral factors, such as diabetes and hypertension. Descriptive analyses incorporated sample weights, and Poisson regression was employed to assess associations between polypharmacy and the independent variables. Analyses were stratified by the five macroregions of Brazil: North, Northeast, Southeast, South, and Central‐West.

**Results:**

The study included 6917 participants aged 50 years or older. Differences in polypharmacy prevalence were observed across Brazilian macroregions. In the Central‐West, polypharmacy was less frequent among rural residents (PR = 0.84; 95% CI: 0.82–0.85) than among urban residents. In the North, polypharmacy was more frequent among non‐white individuals (PR = 1.08; 95% CI: 1.02–1.15) and less frequent among Black individuals (PR = 0.92; 95% CI: 0.88–0.96) compared with white individuals. In the Southeast and South, polypharmacy was more frequent among adults aged 80 years or older (PR = 1.14; 95% CI: 1.08–1.19 and PR = 1.17; 95% CI: 1.08–1.27, respectively) than among younger groups. Although no formal statistical comparisons between regions were performed, the observed estimates and their confidence intervals indicate regional variation in polypharmacy.

**Conclusion:**

This study identified regional disparities in polypharmacy prevalence across Brazil's macroregions, influenced by factors such as age, chronic conditions, and socioeconomic status. Strengthening primary care, promoting rational medication use, addressing inequalities, and integrating prevention strategies are crucial to mitigating its negative impacts.

## Key Points

- Polypharmacy prevalence varies across Brazilian macroregions.
- Older adults (> 80 years) showed the highest prevalence of polypharmacy.
- Diabetes and hypertension are strong predictors of polypharmacy.
- Regional disparities may reflect healthcare access and socioeconomic differences.
- Addressing these disparities is essential to improving elderly care.

## Plain Language Summary

This study examined the use of multiple medications, known as polypharmacy, in Brazilian adults aged 50 and older. Using national data from the ELSI‐Brazil survey, researchers analyzed differences by region and looked at how factors like age, income, education, and chronic diseases such as diabetes and hypertension were linked to polypharmacy. Results showed that older adults and those with chronic conditions were more likely to use many medications, especially in the South and Southeast regions. People in rural areas and with higher education had lower rates. The study highlights the need to improve access to healthcare, promote rational medicine use, and reduce inequalities in different parts of the country to improve health and safety for older people.

## 1 Introduction

Population aging presents complex challenges for health systems, particularly in developing countries [1]. This demographic shift has led to a growing proportion of older adults with healthcare needs that often require long‐term management and specialized care [2]. Consequently, there has been a significant rise in chronic disease prevalence, increasing the demand for continuous care and contributing to higher medication use. Health systems must therefore adapt to the expanding need for long‐term care, integrated services, and sustainable health policies [3].

Polypharmacy is commonly defined as the concurrent use of five or more medications [4]. Among older adults, it is particularly concerning due to its high prevalence, often attributed to the increasing incidence and co‐occurrence of chronic conditions in this population [5]. In high‐income countries, the prevalence of polypharmacy among older adults ranges between 25% and 50%, depending on the definition and population studied [4], while in Brazil it remains less documented and potentially influenced by regional health system disparities. Although the use of multiple medications may be clinically justified in some cases, it significantly raises the risk of adverse effects and drug interactions and may compromise treatment adherence, patient safety, and quality of life [6]. Moreover, excessive or inappropriate medication use is considered a risk factor for frailty and adverse health outcomes [4, 6].

The prevalence and profile of polypharmacy vary substantially depending on demographic, socioeconomic, and educational characteristics, as well as self‐perceived health status [7]. Given the complex interplay among these factors, it is essential for studies to consider regional characteristics and how these variables influence polypharmacy patterns [7, 8]. This is particularly relevant in Brazil, a country with vast territorial extension and marked geographical, social, economic, and cultural disparities [9].

Motivated by these considerations, this study aims to investigate factors associated with polypharmacy prevalence across Brazil's macroregions, using data from the Brazilian Longitudinal Study of Aging (ELSI‐Brazil), which includes sociodemographic, economic, and health data from a nationally representative sample of adults aged 50 years and older. By stratifying analyses across the five macroregions, this study provides new evidence on regional variation in medication use among older adults, contributing to a better understanding of health inequalities and the geographic dimensions of polypharmacy in Brazil. We hypothesized that the prevalence of polypharmacy is higher among older adults with chronic conditions and that socioeconomic and demographic disparities contribute to regional differences in its distribution.

## 2 Methods

### 2.1 Study Design and Population

This study is a cross‐sectional analysis based on secondary data from the second wave (2019–2021) of the Brazilian Longitudinal Study of Aging (ELSI‐Brazil), a study that collects data on the health of Brazilian adults aged 50 and over which [10]. ELSI‐Brazil follows a similar methodology to other longitudinal studies on ageing around the world, allowing for international comparisons. Sample construction was guided by data from the 2010 Demographic Census provided by the Brazilian Institute of Geography and Statistics (IBGE). Stratified sampling was applied, categorizing municipalities by population size. In municipalities with populations of up to 750 000 residents, selection proceeded in three stages (municipality, census tract, and household); in larger municipalities, a two‐stage approach was used (census tract and household). Data were collected from residents across 70 municipalities spanning all Brazilian regions. The final sample of ELSI‐Brazil (2019–2021) consisted of 9849 non‐institutionalized individuals aged 50 and older, representing the national population in this age group [10].

The baseline assessment included four components: household and individual interviews, physical measurements and blood sample collection and analysis. Although all four components were assessed, only the household and individual interview components were used for this study. Further information on the sampling design and methodology employed in ELSI–Brazil can be found elsewhere (http://elsi.cpqrr.fiocruz.br/). The ELSI–Brazil study received approval from the Research Ethics Committee of the René Rachou Institute, Oswaldo Cruz Foundation (CAAE: 34649814.3.0000.5091), and all participants provided informed consent prior to their involvement in the study [10, 11].

### 2.2 Measures

#### 2.2.1 Outcome

The outcome was self‐reported polypharmacy, assessed by the question: “How many regular or continuous medications have you taken in the last two weeks?” Responses were categorized into two groups: up to four medications and five or more medications. Polypharmacy was defined as the use of five or more medications in the last 2 weeks [4].

#### 2.2.2 Exposure Variables

The independent variables included in this study were selected based on the social determinants of health model proposed in previous studies [12, 13]. In addition, sociodemographic variables were considered, including urban/rural residence, sex (male/female), age groups (50–59, 60–69, 70–79, 80 or older), skin color (white, black, and brown), education level (up to 8 years of schooling or more than 8 years of schooling), and per capita income, which was categorized into quintiles. Variables related to health conditions and behavioral factors were also included, such as the diagnosis of diabetes and hypertension, both categorized as ‘yes’ or ‘no’; health insurance status (yes/no); and the use of medical services in the past 12 months (yes/no). This variable was used in a previous study [14]. All health condition variables, including diabetes and hypertension, were self‐reported based on participants’ answers to standardized ELSI‐Brazil questionnaires. Information on health service use and health insurance was also collected through structured interviews.

### 2.3 Statistical Analysis

Data analysis was performed using STATA 14.0 (Stata Corporation, College Station, TX, USA). A descriptive analysis incorporating sampling weights was carried out, followed by an examination of the associations between polypharmacy and socioeconomic and demographic factors using Poisson regression. To take account of the complex sampling design, the data was weighted, and the design effect was incorporated using *Survey Data Analysis* in STATA. Moreover, the goodness‐of‐fit was assessed to ensure a reliable fit of the data to Poisson's model. Adjusted prevalence ratios (PR) were calculated, with a 5% significance threshold. All analyses were stratified by the five geographic macroregions of Brazil: North, Northeast, Southeast, South, and Central‐West. PR and corresponding 95% confidence intervals (95% CI) were estimated using Poisson regression models with robust variance.

Missing data were identified and coded prior to analysis. Complete case analysis was then performed, assuming that missingness occurred at random, as is generally assumed in population‐based epidemiological surveys such as ELSI‐Brazil. The inclusion of independent variables in the adjusted models was guided by theoretical and empirical relevance, following the conceptual framework of social determinants of health [12, 13] and previous studies addressing factors associated with polypharmacy [4, 14]. This strategy aimed to minimize residual confounding and enhance interpretability of associations.

## 3 Results

The final sample included 6917 participants who responded to the analyzed outcome. Table 1 shows the descriptive data and the proportions of polypharmacy among Brazilian adults, highlighting variations between gender, skin color, schooling and chronic conditions such as diabetes and hypertension. The highest proportion of polypharmacy was in individuals with lower levels of education (78.0%) with up to 8 years of study and in those diagnosed with hypertension (74.7%). In contrast, lower proportions were observed in individuals with health insurance (23.8%) and in those who used medical services in the last 12 months (90.3%).

**TABLE 1:** Descriptive analysis and proportions of polypharmacy by socioeconomic and demographic variables in Brazilian adults (*n* = 6917).

Variables	*n* (%)^b^	Proportion of polypharmacy by socioeconomic and demographic factors (95% CI)^a^, ^b^
Socioeconomic and demographic factors		
Region		
North	6.6	02.9 (0.1–07.3)
Northeast	28.1	20.2 (13.7–28.6)
Southeast	43.2	52.8 (41.5–63.9)
South	13.3	15.0 (08.7–24.8)
Central‐West	8.6	08.8 (03.9–18.9)
Zone		
Rural	84.4	11.3 (0.3–15.0)
Urban	15.5	88.6 (84.9–91.6)
Sex		
Male	54.3	37.7 (35.3–40.2)
Female	45.6	62.2 (59.7–64.6)
Skin color		
White	46.6	51.5 (46.4–56.6)
Black	10.8	11.0 (0.9–15.3)
Brown	42.4	36.5 (32.2–41.0)
Per capita income		
Q1	21.8	18.8 (15.5–22.7)
Q2	20.3	19.3 (17.7–20.9)
Q3	17.4	19.8 (17.3–22.5)
Q4	21.0	23.4 (20.7–26.4)
Q5	19.2	18.5 (15.6–21.7)
Education level		
Up to 8 years	71.6	78.0 (75.4–80.3)
More than 8 years	28.4	21.9 (19.6–24.5)
Diabetes diagnosis		
No	82.3	58.9 (55.5–62.2)
Yes	17.6	41.0 (37.7–44.4)
Hypertension diagnosis		
No	50.9	25.2 (21.3–29.6)
Yes	49.0	74.7 (70.3–78.6)
Health insurance		
No	80.3	76.1 (72.7–79.2)
Yes	19.6	23.8 (20.7–27.2)
Use of medical services in the last 12 months		
No	25.3	09.6 (07.6–12.2)
Yes	74.6	90.3 (87.7–92.3)

^a^ 95% CI, 95% confidence interval.

^b^ Accounting for the sample weight.

Table 2 presents the results of the Poisson regression for socioeconomic and demographic factors, including unadjusted and adjusted analyses. In the adjusted analysis, significant associations were found for the Southeast region (PR = 2.29, [1.64–3.19]), South region (PR = 2.55, [1.76–3.68]), Central‐West region (PR = 2.08, [1.42–3.05]) and rural area (PR = 0.90, [0.81–1.00]). Furthermore, age 70–79 (PR = 1.33, [1.18–1.50]), diagnosis of diabetes (PR = 2.08, [1.93–2.24]), diagnosis of hypertension (PR = 1.39, [1.20–1.60]) and use of medical services in the last 12 months (PR = 1.65, [1.40–1.95]) showed a higher prevalence ratio being associated with polypharmacy.

**TABLE 2:** Association between socioeconomic and demographic factors through Poisson regression with robust variance.

Socioeconomic and demographic factors	Unadjusted analysis RP (95% CI)^a^	Adjusted analysis RP (95% CI)^b^
Region		
North	1	1
Northeast	1.35 (0.95–1.93)	1.52 (1.09–2.12)*
Southeast	2.10 (1.50–2.94)*	2.29 (1.64–3.19)*
South	1.03 (1.41–2.94)*	2.55 (1.76–3.68)*
Central‐West	1.82 (1.24–2.68)*	2.08 (1.42–3.05)*
Zone		
Urban	1	1
Rural	0.72 (0.64–0.82)*	0.90 (0.81–1.00)
Sex		
Female	1	1
Male	0.90 (0.83–0.97)*	0.91 (0.83–1.00)
Skin color		
White	1	1
Brown	0.99 (0.86–1.14)	1.00 (0.88–1.14)
Black	0.81 (0.75–0.88)*	0.91 (0.83–1.00)
Age		
50–59	1	1
60–69	1.16 (1.06–1.27)*	1.08 (0.98–1.19)
70–79	1.51 (1.35–1.68)*	1.33 (1.18–1.50)*
80 ou mais	1.52 (1.31–1.77)*	1.39 (1.23–1.58)*
Per capita income		
Q1	1	1
Q2	1.05 (0.92–1.20)*	0.99 (0.89–1.17)
Q3	1.20 (1.03–1.39)*	1.12 (0.96–1.30)
Q4	1.19 (1.02–1.38)*	1.08 (0.93–1.26)
Q5	1.05 (0.90–1.22)*	0.90 (0.76–1.05)
Education level		
Up to 8 years	1	1
More than 8 years	0.76 (0.69–0.84)*	0.84 (0.76–0.94)*
Diabetes diagnosis		
No	1	1
Yes	2.11 (1.96–2.28)*	2.08 (1.93–2.24)*
Hypertension diagnosis		
No	1	1
Yes	1.43 (1.22–1.67)*	1.39 (1.20–1.60)*
Health insurance		
No	1	1
Yes	1.09 (0.98–1.21)	1.08 (0.98–1.20)
Use of medical services in the last 12 months		
No	1	1
Yes	1.79 (1.52–2.11)*	1.65 (1.40–1.95)*

^a^ Unadjusted analysis.

^b^ Adjusted analysis: área, gender, skin color, income, education level.

* *p* < 0.05.

Table 3 presents the proportions of polypharmacy by socioeconomic and demographic factors, stratified by Brazilian macroregions. Rural areas in the Southeast (27.7%, [23.5–32.2]) and South (28.1%, [21.5–35.9]) had the highest proportions of polypharmacy, while urban areas, mainly in the North (5.4%, [1.4–18.6]) and Central‐West (4.7%, [−]), had relatively lower proportions. The proportions of polypharmacy were notably higher among individuals aged 80 years or older, particularly in the South (46.8%, [26.9–67.8]) and Southeast (41.8%, [35.0–49.0]). Individuals with diabetes or hypertension had higher proportions of polypharmacy, especially in the Southeast (45.4%, [37.7–53.4] for diabetes and 32.3%, [28.4–36.3] for hypertension) and in the South (46.9%, [36.4–57.7] for diabetes). Higher proportions of polypharmacy were observed among black individuals in the Southeast (33.9%, [24.0–45.4]) and brown individuals in the South (30.7%, [21.9–41.1]). Furthermore, individuals with health insurance reported higher proportions of polypharmacy, particularly in the Midwest (31.2%, [23.7–39.9]). Some CI overlapped, suggesting that the observed differences between groups may not be statistically significant.

**TABLE 3:** Descriptive analysis and proportions of polypharmacy (use of five or more medications) by socioeconomic and demographic variables in Brazilian adults (*n* = 6917), stratified by macroregion.

Variables		Proportion of polypharmacy by socioeconomic and demographic factors (95% CI)^a^, ^b^				
Socioeconomic and demographic factors	*n* (%)^b^	North (*n* = 6.6%)	Northeast (*n* = 28.1%)	Southeast (*n* = 43.2%)	South (*n* = 13.3%)	Central‐West (*n* = 8.6%)
Zone						
Urban	84.4	12.7 (0.9–16.2)	18.8 (15.9–22.2)	27.7 (23.5–32.2)	28.1 (21.5–35.9)	25.2 (22.8–27.7)
Rural	15.5	05.4 (1.4–18.6)	11.3 (8.8–14.2)	24.2 (20.8–28.0)	19.3 (18.1–20.5)	4.7 (−)
Sex						
Male	54.3	10.1 (7.4–13.5)	13.9 (11.0–17.5)	27.1 (22.5–32.3)	23.6 (20.5–27.0)	23.1 (19.4–27.2)
Female	45.6	13.1 (9.8–17.3)	18.4 (15.8–21.4)	27.6 (23.6–32.0)	27.8 (20.4–36.5)	24.9 (22.3–27.6)
Age						
50–59	47.2	09.8 (7.3–13.0)	15.2 (11.9–19.1)	20.0 (17.4–22.9)	19.1 (15.6–23.2)	27.4 (22.5–32.8)
60–69	29.1	15.0 (10.8–20.4)	16.5 (13.7–19.8)	28.5 (24.0–33.6)	20.7 (18.0–23.6)	20.9 (17.9–24.4)
70–79	16.1	09.6 (4.8–18.5)	21.1 (16.8–26.2)	35.7 (29.9–41.9)	40.8 (33.4–48.6)	23.8 (19.3–28.9)
80 ou mais	7.5	09.3 (4.7–17.6)	15.2 (11.2–20.3)	41.8 (35.0–49.0)	46.8 (26.9–67.8)	23.7 (18.6–29.8)
Skin color						
White	46.6	12.3 (7.1–20.4)	14.0 (11.4–17.0)	28.9 (24.8–33.3)	25.0 (21.0–29.5)	26.1 (22.1–30.5)
Black	10.8	25.1 (16.5–36.0)	19.9 (16.5–23.8)	33.9 (24.0–45.4)	40.7 (10.5–79.9)	21.8 (18.2–25.9)
Brown	42.4	09.4 (7.0–12.5)	17.2 (14.1–20.8)	23.5 (18.6–29.3)	30.7 (21.9–41.1)	23.3 (20.4–26.5)
Per capita income						
Q1	21.8	09.1 (5.9–13.8)	15.8 (12.4–20.0)	30.7 (25.2–36.9)	29.5 (13.3–53.1)	28.7 (20.8–38.2)
Q2	20.3	06.7 (2.9–14.7)	14.8 (10.8–20.0)	30.1 (23.9–37.1)	32.7 (21.3–46.4)	25.8 (20.9–31.4)
Q3	17.4	09.7 (5.7–14.9)	17.7 (12.5–24.4)	32.4 (26.8–38.6)	24.8 (16.7–35.2)	24.3 (21.9–26.9)
Q4	21.0	19.1 (14.8–24.3)	17.8 (14.0–22.3)	25.4 (20.7–30.7)	26.6 (17.7–38.0)	28.3 (24.8–32.2)
Q5	19.2	06.3 (0.9–32.9)	20.1 (14.9–26.6)	22.3 (16.5–29.4)	23.7 (19.0–29.2)	14.2 (8.7–22.2)
Education level						
Up to 8 years	71.6	09 (5.8–14.0)	15.8 (13.7–18.3)	30.1 (25.8–34.7)	29.4 (21.5–38.8)	26.6 (24.0–29.3)
More than 8 years	28.4	16.5 (14.3–19.0)	19.4 (14.6–25.3)	19.4 (15.5–23.9)	16.9 (8.9–29.7)	18.6 (14.1–24.0)
Diabetes diagnosis						
No	82.3	05.6 (03.8–08.3)	11.3 (9.1–14.0)	20.9 (17.9–24.3)	22.0 (16.2–29.1)	17.9 (15.5–20.7)
Yes	17.6	25.0 (20.1–30.5)	33.3 (28.2–38.8)	45.4 (37.7–53.4)	46.9 (36.4–57.7)	45.0 (40.0–50.0)
Hypertension diagnosis						
No	50.9	08.1 (05.7–11.4)	10.5 (6.1–17.6)	17.5 (12.9–23.3)	26.7 (15.9–41.3)	21.1 (17.2–25.6)
Yes	49.0	13.3 (09.9–17.6)	19.0 (16.5–21.7)	32.3 (28.4–36.3)	25.6 (22.9–28.5)	25.5 (22.7–28.4)
Health insurance						
No	80.3	09.8 (06.8–14.0)	16.2 (13.7–19.1)	27.9 (24.1–32.1)	25.7 (20.9–31.2)	22.0 (20.4–23.6)
Yes	19.6	18.7 (15.4–22.5)	20.5 (15.3–27.0)	26.0 (21.3–31.3)	28.1 (18.8–39.7)	31.2 (23.7–39.9)
Use of medical services in the last 12 months						
No	25.3	0	5.4 (2.8–10.3)	16.8 (12.3–22.5)	11.7 (5.7–22.5)	8.4 (6.8–10.3)
Yes	74.6	13.2 (10.4–16.6)	18.4 (16.0–21.0)	28.9 (24.3–33.9)	28.6 (24.3–33.4)	27.5 (24.8–30.4)

*Note:* (−) Not estimable due to single‐PSU strata or insufficient sample size within the subgroup under the survey design.

^a^ 95% CI, 95% confidence interval.

^b^ Taking into account the sample weight.

**FIGURE 1:**
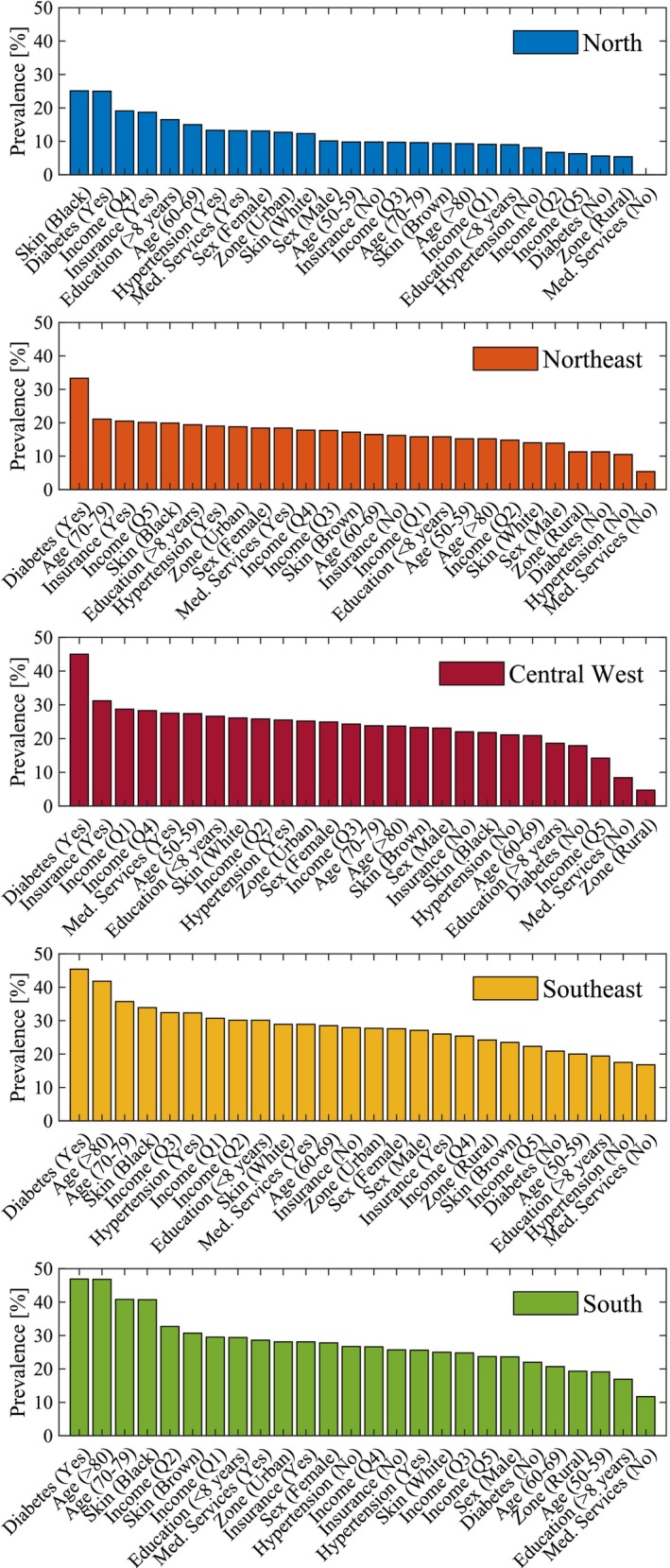
Prevalence ranking of polypharmacy according to socioeconomic and demographic variables across Brazilian macroregions. The figure illustrates the adjusted prevalence ratios (PR) derived from Poisson regression models, highlighting differences in polypharmacy among subgroups stratified by region. Variables include age group, skin color, educational level, presence of diabetes and hypertension, urban/rural residence, and use of health services.

Table 4 presents Poisson regression analyses with robust variance for the associations between socioeconomic and demographic factors and polypharmacy proportions in the five Brazilian macroregions. The adjusted PR and 95% CI are provided. Rural residents in the Central‐West region had a significantly lower polypharmacy prevalence ratio (PR = 0.84, [0.82–0.85]) compared to urban residents, suggesting that rural living is associated with a lower likelihood of polypharmacy in this region. In the North region, mixed‐race individuals had a higher polypharmacy prevalence ratio (PR = 1.08, [1.02–1.15]) compared to white individuals, while black individuals had a lower prevalence ratio (PR = 0.92, [0.88–0.96]) compared to white individuals. Age was significantly associated with polypharmacy, with individuals aged 80 years or older showing higher prevalence ratios in the Southeast (PR = 1.14, [1.08–1.19]) and South (PR = 1.17, [1.08–1.27]) regions compared to the reference group (50–59 years). Similarly, individuals aged 70–79 years in the Southeast (PR = 1.08, [1.03–1.14]) and South (PR = 1.11, [1.05–1.18]) regions had higher polypharmacy PR compared to the reference group. Regarding education level, individuals with more than 8 years of schooling in the Central‐West region had a lower polypharmacy prevalence ratio (PR = 0.94, [0.89–0.99]) compared to those with 8 or fewer years of schooling. This indicates that higher education is associated with a lower likelihood of polypharmacy in this region. Diabetes was consistently associated with higher polypharmacy PR across all regions. Prevalence rates ranged from (PR = 1.19 [1.13–1.24]) in the North to (PR = 1.22 [1.18–1.27]) in the Central‐West region, highlighting a strong and consistent association between diabetes and polypharmacy (Figure 1). Although formal statistical comparisons between regions were not performed, observed differences in adjusted PR, particularly where CI did not overlap, indicate the presence of regional variation in polypharmacy.

**TABLE 4:** Association between socioeconomic and demographic factors by Brazilian macroregions using Poisson regression with robust variance.

Adjusted Analysis—Prevalence ratio (95% CI)^a^					
	North PR (95% CI)	Northeast PR (95% CI)	Southeast PR (95% CI)	South PR (95% CI)	Central‐West PR (95% CI)
Zone					
Urban	1	1	1	1	1
Rural	0.98 (0.92–1.05)	0.96 (0.93–0.98)*	0.97 (0.94–1.00)	0.99 (0.95–1.04)	0.84 (0.82–0.85)*
Sex					
Female	1	1	1	1	1
Male	0.94 (0.91–0.96)	0.97 (0.94–1.00)	0.99 (0.96–1.03)	0.99 (0.95–1.03)	1.00 (0.97–1.0)
Skin color					
White	1	1	1	1	1
Brown	1.08 (1.02–1.15)*	1.04 (0.99–1.08)	1.00 (0.94–1.05)	1.05 (0.85–1.29)	0.96 (0.92–1.00)
Black	0.92 (0.88–0.96)*	1.02 (0.99–1.06)	0.96 (0.93–1.00)	1.03 (0.93–1.13)	0.97 (0.93–1.00)
Age					
50–59	1	1	1	1	11
60–69	0.98 (0.93–1.03)	1.00 (0.96–1.04)	1.04 (1.01–1.07)*	0.99 (0.94–1.05)	0.91 (0.87–0.96)*
70–79	0.98 (0.91–1.05)	1.04 (1.00–1.08)*	1.08 (1.03–1.14)*	1.11 (1.05–1.18)*	0.95 (0.91–1.01)
80 ou mais	0.95 (0.90–1.01)	1.00 (0.94–1.06)	1.14 (1.08–1.19)*	1.17 (1.08–1.27)*	0.92 (0.86–0.98)*
Per capita income					
Q1	1	1	1	1	1
Q2	1.01 (0.95–1.08)	1.00 (0.96–1.04)	0.99 (0.92–1.06)	1.00 (0.85–1.16)	0.95 (0.89–1.02)
Q3	1.02 (0.98–1.06)	1.02 (0.95–1.09)	1.00 (0.93–1.07)	0.96 (0.80–1.15)	0.96 (0.91–1.02)
Q4	1.07 (1.02–1.12)*	1.01 (0.95–1.06)	0.97 (0.92–1.02)	0.97 (0.82–1.15)	1.00 (0.94–1.07)
Q5	0.95 (0.89–1.00)	1.02 (0.94–1.09)	0.94 (0.89–0.99)*	0.95 (0.78–1.15)	0.85 (0.78–0.92)*
Education level					
Up to 8 years	1	1	1	1	1
More than 8 years	1.04 (1.01–1.07)*	1.02 (0.96–1.08)	0.96 (0.92–1.00)	0.92 (0.82–1.04)	0.94 (0.89–0.99)*
Diabetes diagnosis					
No	1	1	1	1	1
Yes	1.19 (1.13–1.24)*	1.18 (1.13–1.23)*	1.19 (1.15–1.23)*	1.22 (1.13–1.31)*	1.22 (1.18–1.27)*
Hypertension diagnosis					
No	1	1	1	1	1
Yes	1.04 (0.99–1.09)	1.06 (1.01–1.04)*	1.09 (1.05–1.14)*	0.97 (0.89–1.05)	1.00 (0.96–1.04)
Health insurance					
No	1	1	1	1	1
Yes	0.94 (0.90–0.99)*	1.06 (1.01–1.12)*	1.00 (0.97–1.03)	1.04 (0.99–1.10)	1.12 (1.06–1.18)*
Use of medical services in the last 12 months					
No	1	1	1	1	1
Yes	1.08 (1.03–1.14)*	1.09 (1.04–1.14)*	1.09 (1.05–1.13)*	1.09 (0.98–1.22)	1.14 (1.11–1.17)*

^a^ Adjusted analysis: zone, sex, skin color, age, per capita income, education level, diabetes diagnosis, hypertension diagnosis, health insurance, use of medical services in the last 12 months.

* *p* < 0.05.

## 4 Discussion

The demographic transition poses significant challenges for health systems at a global and local level as populations age, with an increasing number of individuals living longer and having to live with a greater number of chronic diseases [1]. In Brazil, this trend is particularly pronounced due to the rapid ageing of the population and the concomitant increase in chronic diseases, in addition to infectious and external causes (triple burden of disease), which contribute to the high prevalence of polypharmacy among older adults [15]. This study highlights that polypharmacy is especially prevalent among those aged 80 years and older, a group that often deals with chronic conditions such as hypertension and diabetes. These conditions require complex medication regimens, increasing the risks of adverse drug reactions, potentiating drug–drug interactions, and negatively affecting health‐related quality of life in older adults [16].

Regional disparities in the prevalence of polypharmacy were evident in this study, suggesting the role of sociodemographic factors in clinical outcomes and persistent inequalities in access to and utilization of health care services in Brazil [17]. The Southeast and South regions have the highest rates, particularly among older age groups, while the Central‐West region and rural areas have lower prevalence, potentially reflecting barriers to access to health care. Chronic conditions such as diabetes and hypertension emerge as consistent determinants of polypharmacy across all regions. Furthermore, disparities in educational level and income may suggest the role of socioeconomic inequalities in shaping access to medicines and health services. These patterns are consistent with findings from other Latin American and middle‐income countries, where aging populations and social inequalities contribute to differences in medication use and access to care. These findings highlight the importance of considering regional and individual determinants for the development of interventions to manage polypharmacy in Brazil [5, 7].

Given these disparities, targeted interventions are crucial to ensure equitable access to medications and healthcare services [3]. To comprehensively address polypharmacy in the Brazilian context, it is essential to implement evidence‐based strategies that consider the specific characteristics of the country's macro‐regions [15, 18]. A regionalized and equity‐oriented approach should guide actions that integrate primary health care and rational pharmacotherapy, avoiding redundancy in policy recommendations while emphasizing their combined role in reducing inequalities. Regular prescription review programs and the strengthening of primary healthcare services, focusing on the integrated management of chronic conditions, can play a significant role [19, 20]. Additionally, preventive measures and health promotion strategies targeting modifiable risk factors such as unhealthy diets, physical inactivity, and tobacco use are crucial to reducing the burden of chronic diseases that drive polypharmacy [21]. Health education initiatives should empower individuals to take greater control of their health by adopting healthier lifestyles and encourage discussions about treatment goals with healthcare providers [22, 23].

The study has some limitations. Information on diabetes, hypertension, use of health services, and health insurance was self‐reported, which may have introduced information bias due to recall or reporting errors. Nevertheless, these variables were collected through standardized questionnaires by trained interviewers, reducing the likelihood of systematic misclassification. Another limitation concerns missing data. Although missingness was assumed to occur at random and handled by complete case analysis, this may have slightly reduced statistical power, a common limitation in population‐based surveys. The cross‐sectional design also does not allow for causal inferences between polypharmacy and associated factors. In addition, the reliance on secondary data may limit control over the data collection processes. However, the use of a nationally representative sample increases the generalizability of the study, providing valuable information on the epidemiological characteristics of polypharmacy in Brazil, a country with significant regional diversity.

Understanding these dynamics is essential for developing effective and context‐specific strategies to address polypharmacy and its implications for the health of older adults in Brazil. Strengthening primary healthcare and promoting rational use of medicines, while integrating prevention and health promotion, are essential to mitigate the risks associated with polypharmacy and to advance equity in access to care.

## Author Contributions

Conception and design of study: Clodoaldo Antônio De Sá, Orlando Luiz Do Amaral Juniot, Thiago André Carniel, Fátima Kremer Ferretti, and Vanessa da Silva Corralo. Analysis and/or interpretation of data: Clodoaldo Antônio De Sá, Orlando Luiz do Amaral Júnior, and Thiago André Carniel. Drafting the manuscript: Clodoaldo Antônio De Sá, Orlando Luiz do Amaral Júnior, Thiago André Carniel, Fátima Kremer Ferretti, Vanessa da Silva Corralo. Approval of the version of the manuscript to be published: Clodoaldo Antônio De Sá, Orlando Luiz do Amaral Júnior, Thiago André Carniel, Fátima Kremer Ferretti, and Vanessa da Silva Corralo.

## Funding

The authors have nothing to report.

## Conflicts of Interest

The authors declare no conflicts of interest.

## Supporting information


**Data S1:** STROBE Statement—checklist of items that should be included in reports of observational studies.

## Data Availability

Data will be made available upon request to the corresponding author.
