# Supplementary material for: Association Between Polypharmacy and Socioeconomic and Demographic Factors in Adults Aged 50 Years and Older by Brazilian Macroregions
Source: Pharmacoepidemiol Drug Saf. 2026 Jan 30;35(2):e70307. doi: 10.1002/pds.70307 (PMC12859391; doi:10.1002/pds.70307)
Supplement: Supplementary file 1 — Data S1: STROBE Statement—checklist of items that should be included in reports of observational studies. [file PDS-35-e70307-s001.docx]

STROBE Statement—checklist of items that should be included in reports of observational studies.

| **Item** | **Recommendation** | **Relevant excerpt (from manuscript)** | **Section / Paragraph** |
| --- | --- | --- | --- |
| **Title and Abstract** |  |  |  |
| 1(a) | Indicate the study design with a commonly used term in the title or abstract | “A cross-sectional analysis was conducted using data from the second wave (2019–2021) of ELSI-Brazil.” | Title and Abstract |
| 1(b) | Provide an informative and balanced summary of what was done and what was found | Abstract presents objectives, methods, main results, and conclusions. | Abstract |
| **Introduction** |  |  |  |
| 2 | Explain the scientific background and rationale | “Population aging presents complex challenges for health systems, particularly in developing countries...” | Introduction, paragraphs 1–3 |
| 3 | State specific objectives, including any prespecified hypotheses | “We hypothesized that the prevalence of polypharmacy is higher among older adults with chronic conditions and that socioeconomic and demographic disparities contribute to regional differences in its distribution.” | Introduction, final paragraph |
| **Methods** |  |  |  |
| 4 | Present key elements of the study design early in the paper | “This study is a cross-sectional analysis based on secondary data from the second wave (2019–2021) of the Brazilian Longitudinal Study of Aging (ELSI-Brazil).” | Methods – *Study design and population*, paragraph 1 |
| 5 | Describe the setting, locations, and relevant dates | “Data were collected from residents across 70 municipalities spanning all Brazilian regions… 2019–2021.” | Methods – *Study design and population*, paragraph 2 |
| 6(a) | Cross-sectional study—Give eligibility criteria and sources/methods of participant selection | “The final sample of ELSI-Brazil (2019–2021) consisted of 9,849 non-institutionalized individuals aged 50 and older...” | Methods – *Study design and population*, paragraph 3 |
| 7 | Clearly define all outcomes, exposures, predictors, and confounders | “The outcome was self-reported polypharmacy… categorized into two groups: up to four medications and five or more medications.” | Methods – *Outcome* |
| 8 | For each variable, give data sources and methods of assessment | “All health condition variables, including diabetes and hypertension, were self-reported based on participants’ answers to standardized ELSI-Brazil questionnaires.” | Methods – *Exposure variables*, final paragraph |
| 9 | Describe any efforts to address potential sources of bias | “Missing data were identified and coded prior to analysis… complete case analysis… inclusion of variables guided by theoretical and empirical relevance.” | Methods – *Statistical analysis*, paragraph 2 |
| 10 | Explain how the study size was determined | “The final sample included 6,917 participants who responded to the analyzed outcome.” | Results, first paragraph |
| 11 | Explain handling of quantitative variables | “Responses were categorized into two groups: up to four medications and five or more medications.” | Methods – *Outcome* |
| 12(a) | Describe all statistical methods, including those used to control for confounding | “Poisson regression was employed… Adjusted prevalence ratios (PR) were calculated, with a 5% significance threshold.” | Methods – *Statistical analysis*, paragraph 1 |
| 12(b) | Describe methods used to examine subgroups and interactions | “Analyses were stratified by the five geographic macroregions of Brazil: North, Northeast, Southeast, South, and Central-West.” | Methods – *Statistical analysis*, end of paragraph 1 |
| 12(c) | Explain how missing data were addressed | “Missing data were identified and coded prior to analysis. Complete case analysis was then performed, assuming that missingness occurred at random.” | Methods – *Statistical analysis*, paragraph 2 |
| 12(d) | Describe analytical methods accounting for sampling strategy | “To take account of the complex sampling design, the data was weighted, and the design effect was incorporated using Survey Data Analysis in STATA.” | Methods – *Statistical analysis*, paragraph 1 |
| 12(e) | Describe any sensitivity analyses | Not performed; acknowledged as a limitation (“absence of sensitivity analyses”). | Discussion, paragraph 4 |
| **Results** |  |  |  |
| 13(a) | Report numbers of individuals at each stage | “The final sample included 6,917 participants...” | Results, first paragraph |
| 13(b) | Give reasons for non-participation | Not applicable (secondary data from ELSI-Brazil; details in references 10–11). | — |
| 13(c) | Consider use of a flow diagram | Not used; sampling described in text. | — |
| 14(a) | Give characteristics of participants and confounders | “Table 1 shows the descriptive data and proportions of polypharmacy among Brazilian adults…” | Results, first paragraph; Table 1 |
| 14(b) | Indicate number of participants with missing data | “Complete case analysis was then performed…” | Methods – *Statistical analysis*, paragraph 2 |
| 15 | Report numbers of outcome events or summary measures | Results presented as proportions and prevalence ratios in Tables 1–4. | Results section |
| 16(a) | Give unadjusted and adjusted estimates and precision (95% CIs) | “Table 2 presents… unadjusted and adjusted analyses… Table 4 presents Poisson regression analyses with robust variance.” | Results, paragraphs 2–4; Tables 2 and 4 |
| 16(b) | Report category boundaries when variables were categorized | “Age groups (50–59, 60–69, 70–79, 80 or older), and per capita income categorized into quintiles.” | Methods – *Exposure variables*, paragraph 2 |
| 17 | Report subgroup or stratified analyses | “Analyses were stratified by the five geographic macroregions…” | Methods – *Statistical analysis*, end of paragraph 1; Results, paragraphs 3–4 |
| **Discussion** |  |  |  |
| 18 | Summarise key results with reference to objectives | “This study highlights that polypharmacy is especially prevalent among those aged 80 years and older…” | Discussion, paragraph 1 |
| 19 | Discuss limitations, bias, and imprecision | “Information on diabetes, hypertension, use of health services, and health insurance was self-reported… which may have introduced information bias…” | Discussion, paragraph 4 |
| 20 | Overall interpretation considering objectives and evidence | “Understanding these dynamics is essential for developing effective and context-specific strategies to address polypharmacy…” | Discussion, final paragraph |
| 21 | Discuss generalisability of results | “The use of a nationally representative sample increases the generalizability of the study…” | Discussion, paragraph 4 |
| **Other Information** |  |  |  |
| 22 | Give source of funding and role of funders | Funding and institutional information listed under “Affiliation” and “Corresponding author.” | Title page |
